# Acupuncture therapy for radiotherapy-induced adverse effect: A systematic review and network meta-analysis

**DOI:** 10.3389/fpubh.2022.1026971

**Published:** 2022-12-15

**Authors:** Tong Wu, Chengwei Fu, Yiran Deng, Wanping Huang, Jieyu Wang, Yang Jiao

**Affiliations:** ^1^Hubei Provincial Hospital of Traditional Chinese Medicine, Wuhan, China; ^2^Affiliated Hospital of Hubei University of Traditional Chinese Medicine, Wuhan, China; ^3^Institute of Science, Technology and Humanities, Shanghai University of Chinese Medicine, Shanghai, China; ^4^The Second Clinical Medical School, Guangzhou University of Chinese Medicine, Guangzhou, China; ^5^Xiangyang Hospital of Traditional Chinese Medicine, Xiangyang, China; ^6^General Hospital of The Yangtze River Shipping, Wuhan, China

**Keywords:** acupuncture therapy, radiotherapy, adverse effect, network meta-analysis, systematic review

## Abstract

**Objective:**

To evaluate the efficacy of different acupuncture therapies for radiotherapy-induced adverse effects (RIAEs) and find out the optimal scheme.

**Methods:**

Eligible randomized controlled trials (RCTs) were collected from inception to June 2020 from 9 bibliographic databases. The risk of bias evaluation of the analyzed literature was carried out using the Cochrane risk-of-bias tool. Network meta-analysis was mainly performed using STATA 14.2 and OpenBUGS 3.2.3 by figuring out the network diagrams, league figures, and SUCRA values.

**Results:**

A total of 41 studies with 3,011 participants reported data suitable for network meta-analysis. There was a low to moderate risk of bias in twenty of the articles. ST36 was the most widely prescribed acupoint. Based on network meta-analysis, four outcome indicators were described, namely, acupuncture + medication ranked first in treating radiation enteritis, moxibustion + medication ranked first in preventing radiotherapy-induced leukopenia, acupuncture + medication ranked first in preventing radioactive oral mucositis, and acupuncture ranked first in improving the stimulated salivary flow rate of radioactive xerostomia.

**Conclusion:**

The findings of the network meta-analysis manifested that acupuncture therapy combined with medication has superiority in most RIAEs, both reducing incidence and relieving symptoms. However, high-quality studies are still needed to provide conclusive evidence.

**Systematic review registration:**

https://inplasy.com/inplasy-2020-7-0054/, identifier: INPLASY202070054.

## Introduction

Radiotherapy plays a vital role in treating cancer. As issued by the WHO, radiotherapy contributed to nearly 40% of curable cancer (1, 2). However, serious adverse effects induced by radiotherapy have become a major concern despite its absolute benefits (3). Common radiotherapy-induced adverse effects (RIAEs) include radiodermatitis, xerostomia, and enteritis in local reactions and leukopenia, fatigue, and anepithymia in systemic reactions (4, 5). Failure to control RIAEs will decrease patients' life quality and even cause treatment withdrawal (6). For instance, most sufferers with xerostomia may complain of difficulty in chewing and swallowing and experience several negative consequences, including increased risk of caries, impaired sleep, and psychological and social disability (7).

Ulrike Hoeller noted three common high-risk factors of RIAE, namely, radiation technique and dose, drugs, and individual radiosensitivity (8), who indicated that radiation resistance could provide a good direction for prevention and treatment. However, some anti-radiation drugs also have many side effects, such as drug resistance, nausea, vomiting, and osteoporosis (9, 10), and even the efficacy of amifostine remains controversial (11). In general, current medications for RIAE are limited by side effects and a lack of proven specificity (11–13). Therefore, better alternatives to reduce RIAE are still urgently needed.

With complementary and alternative therapies frequently used over the past 30 years, acupuncture-related interventions have been employed as a new alternative in the field of RIAEs (14–16). Evidence-based medical studies have recommended acupuncture for patients suffering from xerostomia after radiotherapy (17, 18). In addition, a set of experiments on moxibustion were performed to decrease the gastrointestinal toxicities of radiotherapy (19–21). As various acupuncture-related methods targeted at RIAE gained popularity, clinical practitioners were left confused to select an optimal intervention from abundant acupuncture therapies.

The abovementioned confusion led to studies centering around the efficacy of different acupuncture therapies on RIAEs. Previous systematic reviews provided the results of comparisons between acupuncture and placebo controls (14, 22); however, similar types of evidence are far from adequate for comprehensive guidelines. Therefore, this review is aimed at evaluating the validity of different acupuncture therapies based on data from randomized controlled trials (RCTs) and conducting a merit ranking to find out the optimal scheme by a network meta-analysis (NMA).

## Methods

The review has been registered on INPLASY (http://inplasy.com/) with a registration ID of INPLASY202070054. The Preferred Reporting Items for Systematic Review and Meta-Analysis (PRISMA) statement has been used in the article (23). Appendix 1 displays the PRISMA NMA Checklist. Since this is a systematic literature review, ethical approval is not required. The protocol was published in January 2021 (24).

### Literature search strategy

From inception to October 2022, researchers collected relevant randomized controlled trials (RCTs) from 9 bibliographic databases, including PubMed/Medline, Cochrane Library, Web of Science, Ebsco, Embase, China National Knowledge Infrastructure (CNKI), Wanfang Database, VIP Database, and China Biology Medicine Disc (CBM). The language was restricted to Chinese and English. The specific search strategy of different databases was modified properly. Appendix 2 describes a detailed search strategy.

### Selection

First, two reviewers (Yiran Deng and Wanping Huang) browsed the titles and abstracts independently to exclude studies unrelated to the research theme. Then reviewers took a full-text reading of filtered studies to have an elaborate selection. The third reviewer (Jieyu Wang) made the final judgment if any disagreements existed. In the case of repeated publications, only the latest and the most comprehensive results were obtained.

### Eligibility criteria

Studies were included if the following criteria were met: (1) RCTs in peer-reviewed journals; (2) individuals diagnosed as RIAE or cancer patients received radiotherapy, regardless of the type of cancer; (3) experimental group received acupuncture therapies [i.e., acupuncture, moxibustion, electroacupuncture (EA), acupressure, acupoint injection (AI), acupoint plaster (AP), and transcutaneous electrical stimulation (TEN)] with or without medication; (4) control group contained placebo, usual care, and medication; and (5) at least one of the following outcomes was reported: the response rate of RIAE, the incidence of RIAE, safety evaluation, specific outcome indicators such as salivary flow rate for xerostomia and leukocyte level for leukopenia after radiotherapy.

Studies were excluded if the following criteria were met: (1) non-RCTs; (2) studies without complete acupoint prescriptions; and (3) full text not found or deficient original data.

### Data extraction

After selection, two researchers (Tong Wu and Chengwei Fu) set up a standard extraction excel file and independently extracted literature information, patient information, and outcomes information. The third researcher (Yang Jiao) acted as a referee in the context of ambiguity and divergence. Given that some research results did not demonstrate clear baseline data or required data, the authors calculated the statistical value. Furthermore, GetData Graph Digitizer was used to obtain digital information from figures.

After data extraction, another round of screening was required due to the strict limitations of network meta-analysis. If <5 studies reported a common outcome of the same RIAE or the interventions could not be compared in tandem by the same measures, studies concerning the RIAE would be deleted.

### Risk of bias assessment

The Cochrane risk-of-bias (ROB 2.0) was performed to assess the quality. Evaluation contents were laced with 5 items. Two reviewers (Tong Wu and Chengwei Fu) made a judgment independently, and the third reviewer (Yang Jiao) checked again when any disagreements arose. If a high risk appeared during the 5 items, or more than 2 items were considered as some concerns, the study would be regarded as high risk. If 5 items were all considered low risk, the study would be regarded as low risk. Other circumstances raised some concerns.

### Statistical analysis

Network meta-analysis is the development of pairwise meta-analysis, straddling both direct evidence and indirect evidence. To ensure consistency of evidence, the main characteristics of targeted studies must be similar, which has been stipulated through eligible criteria. In this study, Stata 14.2 and OpenBUGS 3.2.3 were performed together to present the results of NMA. The authors adopted OR and 95% CI to measure the effect value of dichotomous data, such as incidence and response rate. Relatively, SMD and 95% CI were for continuous variable data, such as salivary flow rate and leukocyte level. In cases of extreme results, when the incidence was 0, the authors added 0.5 to both the incidence and sample size artificially.

Stata 14.2, a common statistical analysis software, was used to draw charts. Authors input information on interventions and sample sizes to make network diagrams. Sample sizes were divided equally when a multi-arm RCT arose. If more than 10 studies described the same outcome, a network funnel plot could be drawn to assess the publication bias.

OpenBUGS 3.2.3 conducted a complex Bayesian framework with Markov Chain Monte Carlo operations to estimate the posterior distribution of parameters. The authors utilized 3 Markov chains for 100,000 stimulation iterations with 1 thinning interval and 20,000 tuning iterations. In the whole operation, the totresdev value was used to assess the degree of convergence. League figures indicated the relative effect of multiple interventions. A relevant *p-value* was calculated, and a value of <0.05 indicated a statistically significant difference. The surface under the cumulative ranking curve (SUCRA) value ranked interventions from 0 to 100%. In this study, the closer the value was to 100%, the higher the rank.

## Results

### Study characteristics

**Figure 1:**
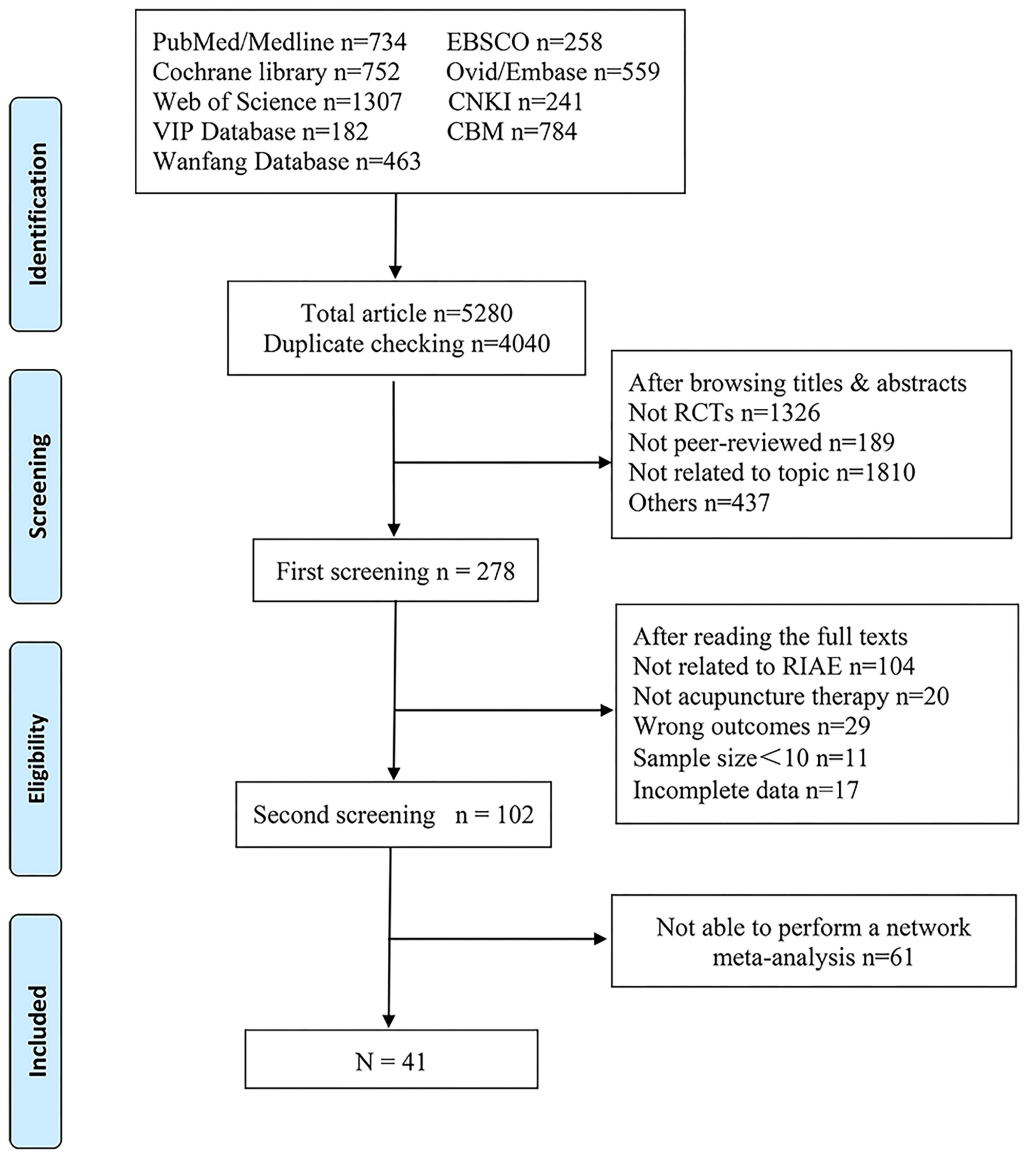
Flowchart of the article selection process.

Figure 1 shows the overall screening process. The authors retrieved 5,280 articles from 9 databases. After deleting the duplicate studies, the authors browsed the titles and abstracts of 4,040 articles and selected 278. A total of 102 articles met the eligibility criteria after reading full texts. Finally, only 41 articles with 3,011 participants, published from 1994 to 2022, reported data suitable for network meta-analysis. More detailed information on these 41 studies is displayed in Table 1. Appendix 3 provides the reference citations.

**Table 1:** Main characteristics of the included studies.

	**Country**	**Tumor location**	**Radiation dosage**	**RIAE**	**Intervention**	**Sample**	**Acupoints**	**Outcome indicator**
Lei, 2021	China	Cervix and prostate and rectum	NA	Radiation enteritis	ACE+ M vs. M	53/53	ST25, RN12, RN4, LR13, ST36, SP6, BL20, BL21, BL25	Clinical response rate
Pan, 2021	China	Cervix and prostate and baldder	NA	Radiation enteritis	Mo+M vs. M	35/35	RN8, RN6, ST36, ST40	Clinical response rate
Wang, 2021	China	Cervix	NA	Radiation enteritis	Mo+M vs. M	43/43	ST25	Clinical response rate
Xie, 2021	China	NA	NA	Radiation enteritis	AI+M vs. M	20/20	SP6, ST37	Clinical response rate
Fang, 2020	China	Pelvic cavity	NA	Radiation enteritis	Mo+M vs. M	43/42	RN8, RN12, RN4, BL25, BL27	Clinical response rate
Dong, 2020	China	Lower abdomen	≥40Gy	Radiation enteritis	Acu+M vs. M	23/23	ST25, RN4, ST37, ST36, BL20, BL21	Clinical response rate
Li, 2019	China	Pelvic cavity and abdomen	NA	Radiation enteritis	AP+M vs. M	20/40	ST36, SP6	Clinical response rate
Yang, 2019	China	Pelvic cavity and abdomen	NA	Radiation enteritis	ACE+M vs. M	40/40	SP6, RN12, RN4, LR13, ST36, ST25, BL20, BL21, BL25	Clinical response rate
Zhang, 2019	China	Pelvic cavity	NA	Radiation enteritis	Acu+M vs. M	44/36	ST36, PC6, BL25	Clinical response rate
Zhong, 2019	China	Pelvic cavity and abdomen	45-60Gy	Radiation enteritis	AI+M vs. M	22/21	SP6, ST37	Clinical response rate
Luo, 2018	China	Pelvic cavity and abdomen	NA	Radiation enteritis	ACE+M vs. M	30/30	ST25, RN12, RN4, LR13, ST36, SP6, BL20, BL21, BL25	Clinical response rate
Chen, 2017	China	Cervix	69-92Gy	Radiation enteritis	Mo+M vs. M	37/38	RN8, RN4, RN6	Clinical response rate
Chen, 2016	China	Cervix	NA	Radiation enteritis	Acu+M vs. M	34/34	ST25, ST37, BL21, BL20, RN4, ST36, SP10, RN8	Clinical response rate
Wu, 2016	China	Cervix	45.0-46.8Gy	Radiation enteritis	Mo+M vs. M	36/42	ST25	Clinical response rate
Long, 2015	China	Cervix	NA	Radiation enteritis	Acu+M vs. M	16/15	BL32, DU1	Clinical response rate
Qiu, 2014	China	Pelvic cavity and abdomen	NA	Radiation enteritis	Mo+Ma+M vs. M	62/62	RN12, RN4, BL25, BL27, RN8	Clinical response rate
Yue, 2015	China	Pelvic cavity and abdomen	NA	Radiation enteritis	Mo+M vs. M	15/15	RN12, RN4, BL25, BL27, RN8	Clinical response rate
Zhu, 2015	China	Pelvic cavity and abdomen	NA	Radiation enteritis	AP+M vs. M	23/23	RN6, RN4, RN8, BL25, ST37, ST39, ST36	Clinical response rate
Qiu, 2015	China	Cervix	56-85Gy	Radiation enteritis	Mo+M vs. M	30/28	RN8, RN4, RN6	Clinical response rate
Lin, 2013	China	Pelvic cavity and abdomen	NA	Radiation enteritis	Mo+M vs. M	63/60	RN12, RN6, RN4, BL25, BL27	Clinical response rate
Ji, 2008	China	Cervix	35-40Gy	Radiation enteritis	Mo+Acu+M vs. M	30/24	ST25, RN4, ST37, ST36, BL20, BL21	Clinical response rate
Li, 2007	China	Rectum	50-70Gy	Radiation enteritis	Mo+Acu+M vs. M	30/30	ST25, RN4, ST37, ST36, BL20, BL21	Clinical response rate
Yang, 1994	China	NA	NA	Radiation enteritis	EA vs. M	30/20	LI11, LI4, ST36, SP6, ST37, ST25, RN6, RN12, KI6, BL57	Clinical response rate
Xie, 2016	China	Lung	60-70Gy	Radiotherapy-induced leukopenia	Mo+M vs. BC	28/28	RN4, RN6, ST36	Incidence rate of RIAE
Zheng, 2014	China	Esophagus	60Gy	Radiotherapy-induced leukopenia	Acu+M vs. BC	30/30	RN22, RN17, PC6, ST36, RN12, SP4	Incidence rate of RIAE
Ge, 2012	China	NA	NA	Radiotherapy-induced leukopenia	AP vs. BC	46/44	BL20, BL17, ST36	Incidence rate of RIAE
Zhu, 2009	China	NA	52-64Gy	Radiotherapy-induced leukopenia	AP vs. M	48/48	ST36, SP10, BL17, DU14, BL20	Incidence rate of RIAE
Zhang, 2007	China	NA	NA	Radiotherapy-induced leukopenia	Ac+M vs. M	80/80	PC6, LI11, ST44, ST36	Incidence rate of RIAE
Sun, 2019	China	Head and neck	60-70Gy	Radioactive oral mucositis	Mo vs. BC	24/24	KI1	Incidence rate of RIAE
Liang, 2015	China	Nasopharynx	66-70Gy	Radioactive oral mucositis	Acu+M vs. M	40/40	ST36, ST7, ST6	Incidence rate of RIAE
Wang, 2012	China	Nasopharynx	66-70Gy	Radioactive oral mucositis	Acu+M vs. BC	60/40	ST36, ST7, ST6	Incidence rate of RIAE
Zhong, 2012	China	Nasopharynx	50-70Gy	Radioactive oral mucositis	AuP vs. BC	40/40	CO1, LO2, TG3, CO2, CO14, CO15, TF4, AH6a, AT4	Incidence rate of RIAE
Liu, 2002	China	Nasopharynx	70-77Gy	Radioactive oral mucositis	AP vs. BC	38/15	KI1	Incidence rate of RIAE
Li, 1999	China	Nasopharynx	70-76Gy	Radioactive oral mucositis	AP vs. BC	68/82	KI1	Incidence rate of RIAE
Huang, 2020	China	Nasopharynx	NA	Radioactive xerostomia	Ma+M vs. M	50/50	KI10	SSFR
Dalbem, 2019	Brazil	Head and neck	60-70Gy	Radioactive xerostomia	TEN vs. BC	37/31	ST6, ST5	SSFR
Wong, 2015	Canada	Head and neck	NA	Radioactive xerostomia	TEN vs. M	73/73	LI4, ST36, SP6, RN24	SSFR
Meng a, 2012	China	Nasopharynx	NA	Radioactive xerostomia	Acu vs. BC	40/46	LU7, KI6, RN24	SSFR
Meng b, 2012	America	Nasopharynx	25Gy	Radioactive xerostomia	Acu vs. placebo	11/12	LU7, KI6, RN24	SSFR
Braga, 2011	Brazil	Head and neck	60.4-75.8Gy	Radioactive xerostomia	Acu vs. BC	12/12	ST3, ST4, ST5, ST6, ST7, SI19, GB2, SJ21, ST36, LI4, LI11, KI3, KI5, LR3	SSFR
Blom, 1996	Sweden	Head and neck	50-68Gy	Radioactive xerostomia	Acu vs. placebo	20/18	DU20, ST3, ST6, ST5, LI18, SI17, ST7, LI18, PC6, HT7, ST36, LR3	SSFR

M, medication; Acu, acupuncture; Mo, moxibustion; ACE, acupoint catgut embedding; EA, electroacupuncture; AI, acupoint injection; AP, acupoint plaster; BC, blank control; Acp, acupressure; Au, auricular pressure; TEN, transcutaneous electric nerve stimulation; Plc, placebo.

In this review, four types of RIAEs were described, including radiation enteritis, radiotherapy-induced leukopenia, radioactive xerostomia, and radioactive oral mucositis. In general, despite the fact that these interventions differed between RIAEs, acupuncture combined with medicine has the broadest application. As radiotherapy dosage was supposed to be related to the severity of RIAEs, included articles listing clear dosage reported that a general population received a dose of more than 50 Gy. A total of 20 studies reported the radiation dose. The average sample size was 72.75 ± 33.96 participants. As for acupoint prescription, ST36 was the most common acupoint used in the above 4 RIAEs. In terms of outcome indicators, studies of radiation enteritis presented its clinical response rate, radiotherapy-induced leukopenia and radioactive oral mucositis presented the incidence rate, and radioactive xerostomia presented a stimulated salivary flow rate (SSFR).

### Quality evaluation

Table 2 shows the risk of bias assessment of 41 articles according to the guidance of the ROB 2.0 tool. Reference citations of these articles would be listed in Appendix 3. A low-to-moderate risk of bias was found in twenty of them. Only 5 articles showed a low risk of bias arising from the randomization process. High risk was caused mostly by randomization, deviations from intended interventions, and measurement of outcomes.

**Table 2:** Risk of bias assessment.

**Author/year**	**RP**	**D**	**M**	**MOD**	**RR**	**O**
Lei, 2021	S	S	L	L	L	S
Pan, 2021	S	S	L	L	S	H
Wang, 2021	S	S	L	L	L	S
Xie, 2021	S	S	L	L	L	S
Fang, 2020	S	S	L	L	S	H
Dong, 2020	S	S	L	L	L	S
Li, 2019	L	L	L	L	L	L
Yang, 2019	S	S	L	L	L	S
Zhang, 2019	H	H	L	L	S	H
Zhong, 2019	S	S	L	L	S	H
Luo, 2018	S	S	L	L	L	S
Chen, 2017	S	S	L	L	L	S
Chen, 2016	S	S	L	L	S	H
Wu, 2016	H	H	L	L	S	H
Long, 2015	S	S	L	L	L	S
Qiu, 2015	S	S	L	L	L	S
Yue, 2015	S	S	L	L	S	H
Zhu, 2015	H	H	L	L	S	H
Qiu, 2014	S	S	L	L	L	S
Lin, 2013	S	S	L	L	S	H
Ji, 2008	S	S	L	L	S	H
Li, 2007	S	S	L	L	S	H
Yang, 1994	S	S	L	L	S	H
Xie, 2016	S	S	L	L	L	S
Zheng, 2014	S	S	L	L	L	S
Ge, 2012	S	S	L	S	S	H
Zhu, 2009	S	S	L	L	S	H
Zhang, 2007	S	S	L	S	S	H
Sun, 2019	S	S	L	L	L	S
Liang, 2015	S	S	H	L	L	H
Wang, 2012	S	S	H	L	L	H
Zhong, 2012	S	S	L	L	S	H
Liu, 2002	H	H	L	S	S	H
Li, 1999	S	S	L	S	S	H
Huang, 2020	S	S	L	L	S	H
Dalbem, 2019	L	L	L	L	L	L
Wong, 2015	L	L	L	L	L	L
Meng a, 2012	L	S	L	L	L	S
Meng b, 2012	L	S	L	L	L	S
Braga, 2011	S	S	L	L	L	S
Blom, 1996	S	L	L	L	L	S

RP, bias arising from the randomization process; D, bias due to deviations from intended interventions; M, bias in measurement of the outcome; MOD, bias due to missing outcome data; RR, bias in selection of the reported result; O, overall risk of bias; L, low risk; S, Some Concern; H, High Risk.

### Network meta-analysis

#### Network diagrams

**Figure 2:**
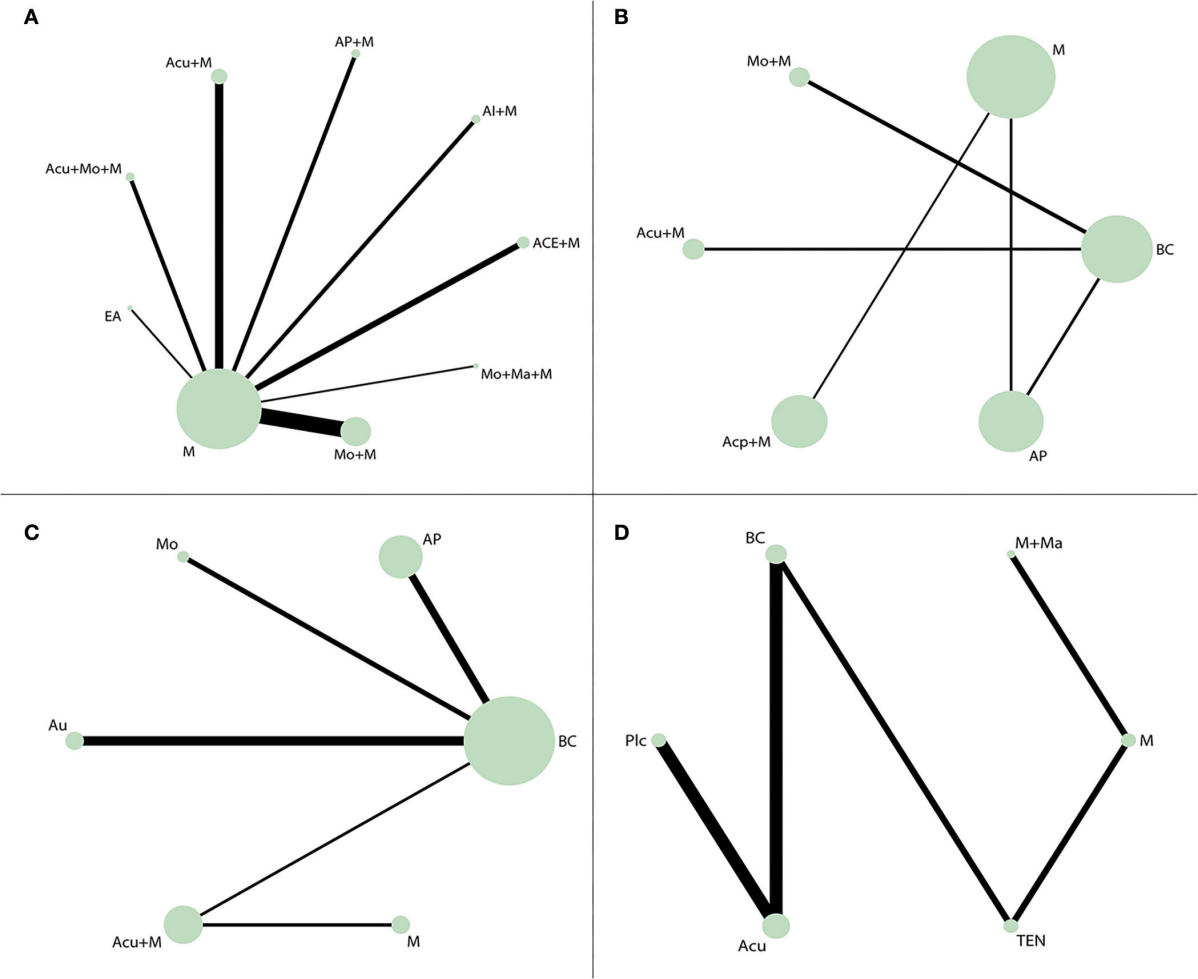
Network diagrams. The size of the node represented the sample size of participants, and the size of the line represented the number of studies comparing the two interventions. **(A)** Network diagrams of response rate of radiation enteritis. A total of 23 studies with 1,553 participants accepted one of 9 interventions, respectively. **(B)** Network diagrams of the incidence rate of radiotherapy-induced leukopenia. A total of 5 studies with 462 participants accepted one of 6 interventions, respectively. **(C)** Network diagrams of the incidence rate of radioactive oral mucositis. A total of 6 studies with 511 participants accepted one of 6 interventions, respectively. **(D)** Network diagrams of SSFR of radioactive xerostomia. A total of 7 studies with 485 participants accepted one of 6 interventions, respectively. Acu, acupuncture; ACE, acupoint catgut embedding; Acp, acupressure; AI, acupoint injection; AP, acupoint plaster; Au, auricular pressure; BC, blank control; EA, electroacupuncture; Plc, placebo; M, medication; Ma, acupoint massage; Mo, moxibustion; TEN, transcutaneous electric nerve stimulation; SSFR, stimulated salivary flow rate.

Network diagrams for interventions in RCTs are shown in Figure 2. Among them, nodes represent various intervention measures, the sizes of nodes represent sample capacity, and the thickness of lines represents the number of studies. No closed loop was displayed. The reduction in the number of studies per comparison increased the uncertainty around the true between-study variance. Generally, combined therapies, such as moxibustion + medication and acupuncture + medication, offered more applications in radiation enteritis. Single therapies such as AP and TEN gained more popularity in the other 3 RIAEs.

#### League figures and SUCRA values

By adjusting the number of iterations, the deviation between the totresdev and the sum of products (research quantum multiplied by the number of arms) was controlled within 10, indicating a good convergence. Results of network analysis were displayed in accordance with the following 4 outcome indicators: response rate of radiation enteritis, incidence rate of radiotherapy-induced leukopenia, incidence rate of radioactive oral mucositis, SSFR of radioactive xerostomia. League figures and SUCRA values supported a possible rank for them.

**Figure 3:**
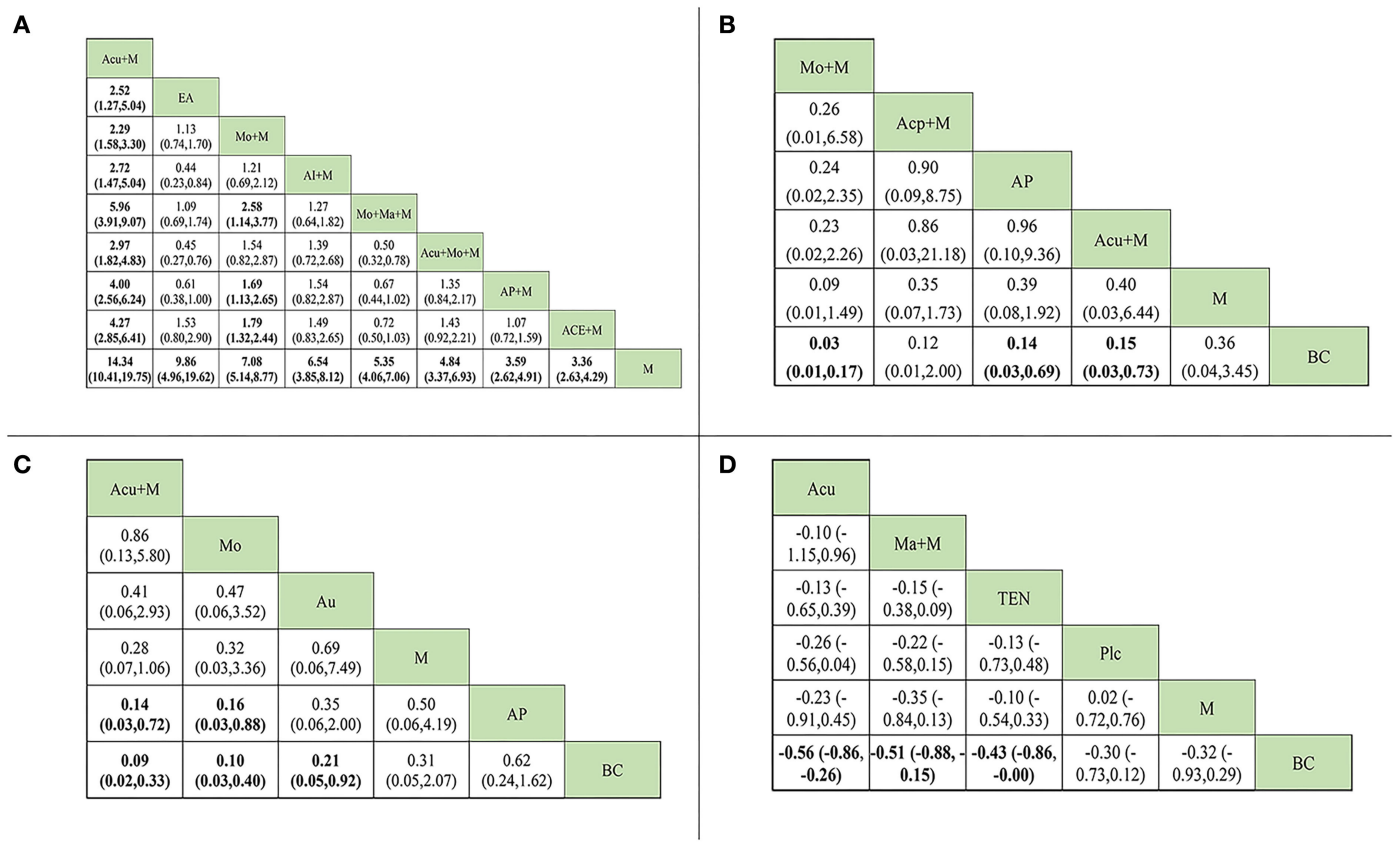
League figure. The data in bold mean the significant difference between the comparison. **(A)** League figure of response rate of radiation enteritis. **(B)** league figure of the incidence rate of radiotherapy-induced leukopenia. **(C)** league figure of the incidence rate of radioactive oral mucositis. **(D)** league figure of SSFR of radioactive xerostomia. Acu, acupuncture; ACE, acupoint catgut embedding; Acp, acupressure; AI, acupoint injection; AP, acupoint plaster; Au, auricular pressure; BC, blank control; EA, electroacupuncture; Plc, placebo; M, medication; Ma, acupoint massage; Mo, moxibustion; TEN, transcutaneous electric nerve stimulation; SSFR, stimulated salivary flow rate.

**Figure 4:**
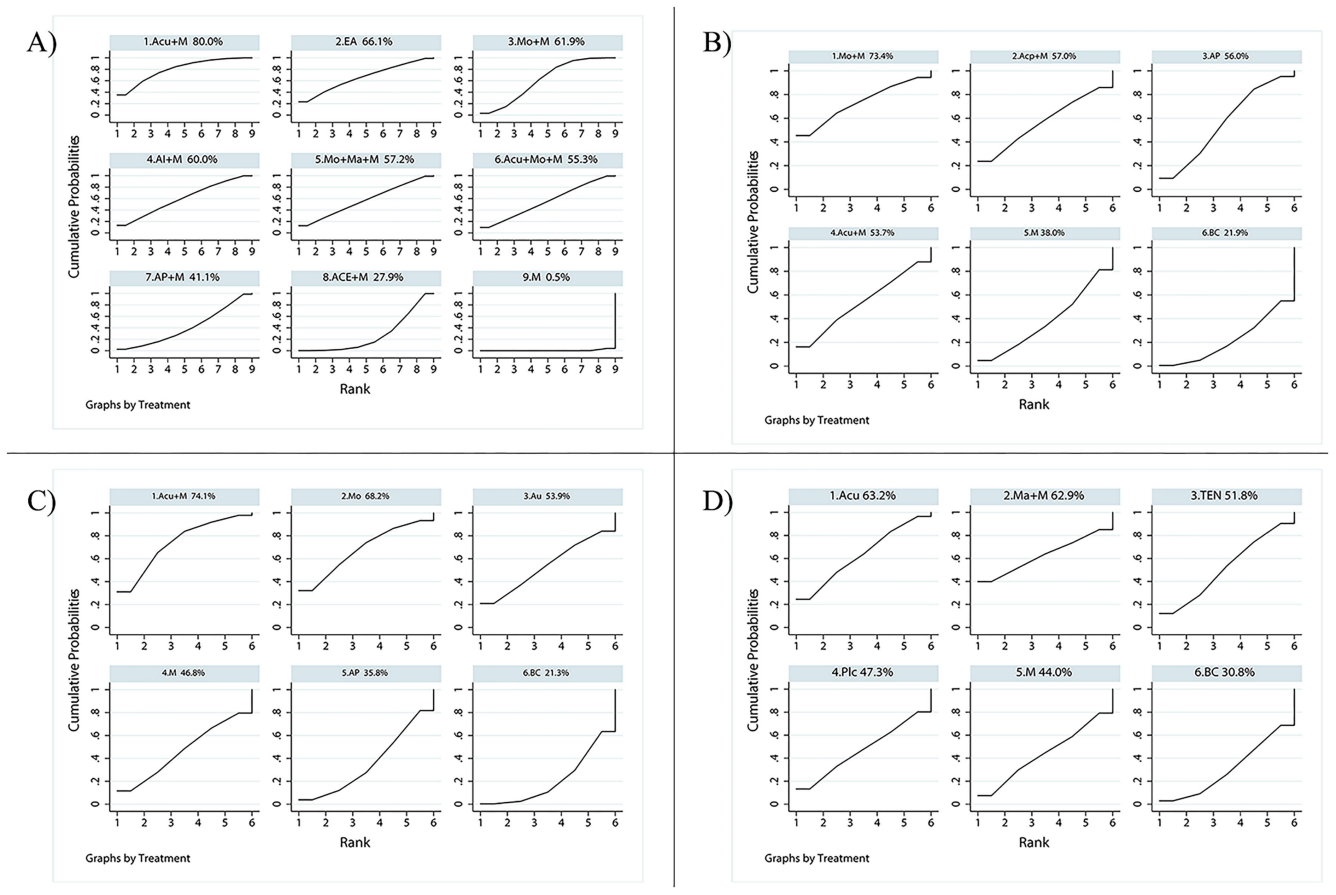
SUCRA value. A bigger SUCRA value represents better efficacy of the intervention. **(A)** SUCRA value of the response rate of radiation enteritis. The rank of each intervention: 1. Acu+M (80.0%) > 2. EA (66.1%) > 3. Mo+M (61.9%) > 4. AI+M (60.0%) > 5. Mo+Ma+M (57.2%) > 6. Acu+Mo+M (55.3%) > 7. AP+M (41.1%) > 8. ACE+M (27.9%) > 9. M (0.5%). **(B)** SUCRA value of the incidence rate of radiotherapy-induced leukopenia. The rank of each intervention: 1. Mo+M (73.4%) > 2. Acp+M (57.0%) > 3. AP (56.0%) > 4. Acu+M (53.7%) > 5. M (38.0%) > 6. BC (21.9%). **(C)** SUCRA value of incidence rate of radioactive oral mucositis. The rank of each intervention: 1. Acu+M (74.1%) > 2. Mo (68.2%) > 3. Au (53.9%) > 4. M (46.8%) > 5. AP (35.8%) > 6. BC (21.3%). **(D)** SUCRA value of SSFR of radioactive xerostomia. The rank of each intervention: 1. Acu (63.2%) > 2. Ma+M (62.9%) > 3. TEN (51.8%) > 4. Plc (47.3%) > 5. M (44.0%) > 6. BC (30.8%). Acu, acupuncture; ACE, acupoint catgut embedding; Acp, acupressure; AI, acupoint injection; AP, acupoint plaster; Au, auricular pressure; BC, blank control; EA, electroacupuncture; Plc, placebo; M, medication; Ma, acupoint massage; Mo, moxibustion; TEN, transcutaneous electric nerve stimulation; SSFR, stimulated salivary flow rate.

From the league figures in Figure 3, a total of 9 interventions were used in treating radiation enteritis, among which combined therapy (the combination between medication and acupuncture, AP, AI, etc.) and the single therapy EA presented a better clinical response rate than medication alone in the control group with a statistical difference. The acupuncture + medication group showed the most superior efficacy (OR = 14.34, CI: 10.41~19.75) compared with the medication alone group and still had a significant difference from other interventions. According to SUCRA values in Figure 4, the acupuncture + medication (SUCRA: 80.0%) group ranked first as the optimal treatment for radiation enteritis.

In the experimental groups, four types of methods were employed for preventing radiotherapy-induced leukopenia. However, its league figure indicated that only 3 of them (moxibution + medication, AP, and acupuncture + medication) had a significant difference from the blank control group, without any statistical difference compared with the medication alone group. Among them, the moxibution + medication group mostly reduced its incidence (OR = 0.03, CI: 0.01~0.17). There was no visible difference among 4 types of methods, but SUCRA values provided a possible rank for reducing incidence and moxibution + medication (SUCRA: 73.4%) ranked first as the optimal prevention.

For radioactive oral mucositis, only one combined therapy (acupuncture + medication) was used. Analyzed studies used single therapies such as moxibustion, auricular pressure, and AP in the experimental groups. Based on the league figure, compared with the blank control group, the lowest incidence came from the combined therapy of acupuncture + medication (OR = 0.09, CI: 0.02~0.33), followed by moxibustion alone (OR = 0.10, CI: 0.03~0.40). Both the combined therapy and moxibustion showed a statistical difference from AP, but not from medication. SUCRA values provided further analysis for possible rankings. Acupuncture + medication performed best, with the highest score (SUCRA: 74.1%).

For radioactive xerostomia, the salivary flow rate was most commonly used as an observation index. These analyzed RCTs used acupuncture, acupoint massage + medication, and TEN as treatments, and all treatments outperformed the blank control group (SMD = −0.56, CI: −0.86~-0.26; SMD = −0.51, CI: −0.88~-0.15; SMD = −0.43, CI: −0.86~-0.00). But they had no significant difference between the placebo and medication. Overall, acupuncture (SUCRA: 63.2%) was still the most favorable intervention for improving SSFR.

#### Network funnel plot

**Figure 5:**
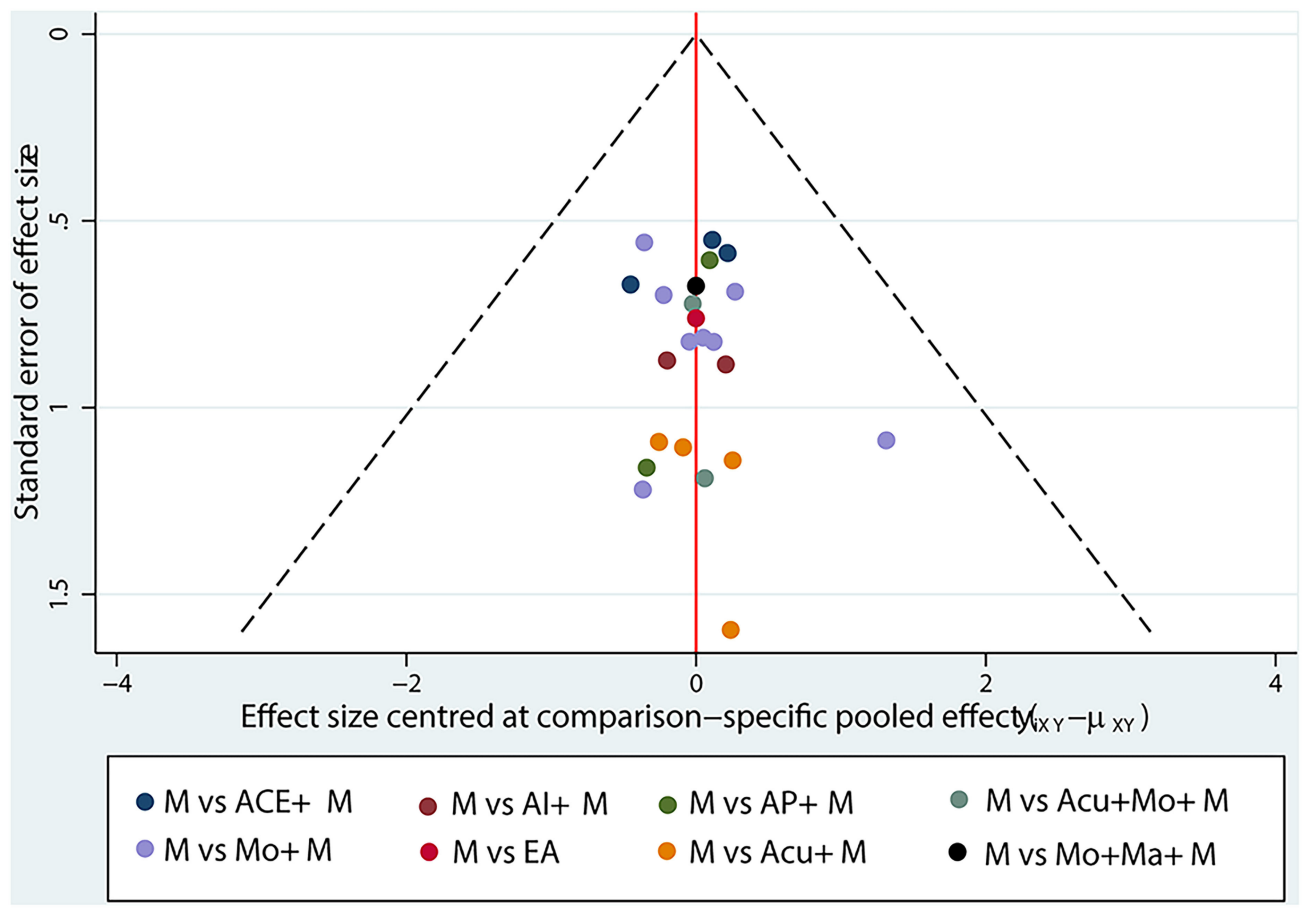
Funnel plot of response rate of radiation enteritis. M, medication; ACE, acupoint catgut embedding; AI, acupoint injection; AP, acupoint plaster; Acu, acupuncture; Mo, moxibustion; EA, electroacupuncture; Ma, acupoint massage.

The study only used the publication bias in the literature related to radiation enteritis. As shown in Figure 5, the horizontal axis represented the aggregate effect, while the vertical axis represented the standard error (SE) of the effect value of Log (OR) in this study. A larger sample size resulted in a smaller SE and higher distribution of the points, and *vice versa*. Ideally, the points representing each study should be evenly distributed on both sides of the middle vertical line. It was basically consistent with 23 RCTs of radiation enteritis, suggesting a low publication bias.

## Discussion

This systematic review involving multiple acupuncture-related treatments might be the first network meta-analysis for RIAEs. Results suggested that the greatest benefits can be achieved using acupuncture + medication for radiation enteritis and radioactive oral mucositis, moxibustion + medication for radiotherapy-induced leukopenia, and acupuncture for radioactive xerostomia.

In this analysis, a total of 23 articles addressed radiation enteritis, suggesting that the application of acupuncture therapies in gastrointestinal function after radiation has been mostly recognized. Regarding the risk of bias assessment, quite a few high-risk studies were attributed to the difficulty in the implementation of blinding. Using acupuncture as an example, the operation process involves communication with patients in order to ensure the curative effect, which is the main reason participants break their blindness (25).

In terms of acupoint prescriptions, the selection principle appeared to be a combination of local and distal acupoints. According to data statistics, ST36 was the most widely used in these 4 RIAEs, which benefits from its powerful regulation effect on the internal environment and homeostasis (26, 27). Former studies have shown that acupoint stimulation in ST36 can present the dual-directional regulation of the neurohumoral system, promote the immune capacity and quality of peripheral blood, and relieve inflammation through several mechanisms such as vagus nerve activation and macrophage polarization (28, 29).

The results of NMA provided some evidence for scheme selection. Acupuncture + medication was reflected as the most effective treatment for radiation enteritis, followed by EA. Acupuncture has gained progressive acceptance in intestinal dysfunction diseases (30, 31). Modern biological studies have proved that acupuncture can activate the signaling pathway in macrophages, which reduces the production of inflammatory cytokines, and also have the potential to control intestinal inflammation, suppress acid secretion *via* different somatic autonomic reflex pathways, and regulate the brain-gut axis through intestinal microbiota (32, 33). When combined with medication, moxibustion also shows a good effect. It might be credited to its effect of warming meridian and inspiring qi of Zang-Fu organs, that is, regulation of the enteric nervous system and the alleviation of visceral hypersensitivity from a biological point of view (34). Both acupuncture and moxibustion can decrease the levels of inflammatory factors in enteric diseases (35). Acupoint catgut embedding (ACE), AI, and AP were also employed combined with medication, respectively. Although these three treatments showed superiority compared with medication alone, more studies on theoretical mechanisms and clinical evidence are still needed. Another noticeable point is that the 2 studies in this analysis used a combination of two kinds of acupuncture therapies (acupuncture + moxibustion or moxibustion + massage) and medication, but they did not behave better as supposed. It may be blamed on an insufficient sample as well as the heterogeneity of duration and stimulation amount. In addition, complex treatments may lead to lower patient compliance, resulting in discounted efficacy and relevant outcome bias.

For leukopenia after radiotherapy, the moxibustion + medication group significantly decreased the incidence, consistent with the results of a previous review (19). In mechanism studies, moxibustion can enhance the activities of serum colony-stimulating factor, which reduces damage toward bone marrow hematopoietic cells caused by radiotherapy and regulate innate immune defensive function as well as improve the tumor immune microenvironment and normalize vascular condition (36). Given that acupuncture + medication did not behave well in preventing radiotherapy-induced leukopenia, authors speculated that powerful reinforcing methods such as moxibustion are more suitable for immune deficiency. In fact, immune deficiency has always been regarded as a deficiency syndrome in TCM, and according to the reinforcing and reducing principle, acupuncture may not be as suitable as moxibustion in such a syndrome. Definitely, these 4 methods (moxibustion + medication, acupoint massage + medication, AP, and acupuncture + medication) did not internally show a significant difference in spite of the sequential order, because the conclusion only based on SUCRA values should be regarded as supportive evidence rather than conclusive evidence (37, 38).

In radioactive oral mucositis, acupuncture combined with medication can mostly prevent it. Similar to intestinal inflammation in radiation enteritis, acupuncture may also have an effect on reducing inflammation of the oral mucosa and reestablishing oral flora balance. However, less relevant mechanism studies were carried out. Besides, auricular pressure as a simple and safe therapy showed a significant difference from the blank control, which can provide a new study point for its clinical prevention (39).

For xerostomia after radiotherapy, studies have proven that acupuncture has definite effectiveness for inhibiting dry mouth and promoting salivary secretion (40, 41). The previous meta-analysis has the same opinion (14, 42). Furthermore, researchers once clearly put forward that acupuncture can attenuate the decrease in salivary immunoglobulin A caused by intense exercise (43). For nearly 30 years, a number of controlled clinical studies on acupuncture for radiation-induced xerostomia have been carried out abroad, and most of the recent results have approved the effectiveness of true acupuncture in treating and improving the life quality of patients (40, 44). In contrast, studies in China prefer to use herb decoction, and only in the past 2 years has acupuncture been applied to xerostomia after radiation. This situation may be influenced by the results of international research, which is a benign academic influence on the development of acupuncture. In the RCTs included, a combination of acupoint massage and medication was also used as treatment and achieved good results. Notably, only one acupoint was stimulated, *viz*. KI10. This point in the kidney meridian of the foot Shaoyin has the fluid characteristic of a He-sea point, so it is quite understandable why it can be used for xerostomia from the theory of TCM. However, no biological mechanism has been studied focused on KI10, which might be a deserving research direction. Besides, a new therapy called TEN appeared, which draws on the meridians and acupoints theory of TCM and endocrine theory. It applies electrode patches on acupoints to excite nerve endings and is often mentioned with EA due to the similarity (45, 46). Wong's study held that the effectiveness of TEN is similar to that of pilocarpine, the most commonly used drug for radioactive xerostomia (47), which conformed to our results. In addition, as a non-invasive treatment, its repeatability and lack of infection have received a great deal of academic attention. Some investigators even believe that TEN may be a safer substitute for EA (45, 48).

Generally speaking, this study is mainly limited by low-quality and inadequate literature. Several RCTs did not clearly report observation time for outcomes and accurate radiation dosage, which also generated heterogeneity. In effect, acupuncture therapy has a variety of parameters, such as timing, acupoint, stimulation intensity, needle technique, electrical waveform, and frequency, inevitably affecting the final data. Due to the neglect of these data in clinical research, it is difficult for us to obtain clues to analyze acupuncture parameters. Future studies would be more data-friendly if critical parameters of acupuncture are recorded, so that subgroup analyses could be carried out, which can provide more reliable evidence. In the process of literature collection, authors found existing studies focused less on RIAEs at different time periods. Considering the close connection between the prognosis of RIAEs and the time window, as well as differences between diagnosis and treatment standards of acute and chronic radioactive reactions, it remains to be studied further.

## Conclusion

The findings of network meta-analysis manifested that acupuncture therapy combined with medication has superiority in most RIAEs, both reducing incidence and relieving symptoms. Among these interventions included in each RIAE, acupuncture + medication was the most effective one for relieving radiation enteritis and preventing radioactive oral mucositis and moxibustion + medication for preventing radiotherapy-induced leukopenia. For patients suffering from radioactive xerostomia, acupuncture performed best in improving their SSFR. However, high-quality studies are still needed to provide conclusive evidence.

## Data availability statement

The original contributions presented in the study are included in the article/Supplementary material, further inquiries can be directed to the corresponding authors.

## Author contributions

TW and CF performed the meta-analysis, wrote the first draft of the manuscript, and contributed equally to this study. JW and YJ supervised the work. YD and WH revised the final manuscript. All authors have read and approved the final manuscript.
