# Supplementary material for: Acupuncture therapy for radiotherapy-induced adverse effect: A systematic review and network meta-analysis
Source: Front Public Health. 2022 Dec 15;10:1026971. doi: 10.3389/fpubh.2022.1026971 (PMC9797977; doi:10.3389/fpubh.2022.1026971)
Supplement: Supplementary file 1 [file Data_Sheet_1.docx]

Appendix 1 PRISMA checklist of the systematic review.

Apeendix 2 Search strategy of

Appendix 3 Reference of included studies

Appendix1

**PRISMA NMA Checklist of Items to Include When Reporting A Systematic Review Involving a Network Meta-analysis**

| **Section/Topic** | **Item #** | **Checklist Item** | **Reported on Page #** |
| --- | --- | --- | --- |
| **TITLE** |  |  |  |
| Title | 1 | Identify the report as a systematic review *incorporating a network meta-analysis (or related form of meta-analysis).* | ***1*** |
|  |  |  |  |
| **ABSTRACT** |  |  |  |
| Structured summary | 2 | Provide a structured summary including, as applicable:  **Background:** main objectives  **Methods:** data sources; study eligibility criteria, participants, and interventions; study appraisal; and *synthesis methods, such as network meta-analysis.*  **Results:** number of studies and participants identified; summary estimates with corresponding confidence/credible intervals; *treatment rankings may also be discussed. Authors may choose to summarize pairwise comparisons against a chosen treatment included in their analyses for brevity.*  **Discussion/Conclusions:** limitations; conclusions and implications of findings.  **Other:** primary source of funding; systematic review registration number with registry name. | 2 |
|  |  |  |  |
| **INTRODUCTION** |  |  |  |
| Rationale | 3 | Describe the rationale for the review in the context of what is already known*, including mention of why a network meta-analysis has been conducted.* | ***4*** |
| Objectives | 4 | Provide an explicit statement of questions being addressed, with reference to participants, interventions, comparisons, outcomes, and study design (PICOS). | 4-5 |
|  |  |  |  |
| **METHODS** |  |  |  |
| Protocol and registration | 5 | Indicate whether a review protocol exists and if and where it can be accessed (e.g., Web address); and, if available, provide registration information, including registration number. | 6 |
| Eligibility criteria | 6 | Specify study characteristics (e.g., PICOS, length of follow-up) and report characteristics (e.g., years considered, language, publication status) used as criteria for eligibility, giving rationale. *Clearly describe eligible treatments included in the treatment network, and note whether any have been clustered or merged into the same node (with justification).* | ***6*** |
| Information sources | 7 | Describe all information sources (e.g., databases with dates of coverage, contact with study authors to identify additional studies) in the search and date last searched. | 6 |
| Search | 8 | Present full electronic search strategy for at least one database, including any limits used, such that it could be repeated. | 6 Appendix 1 |
| Study selection | 9 | State the process for selecting studies (i.e., screening, eligibility, included in systematic review, and, if applicable, included in the meta-analysis). | 6 |
| Data collection process | 10 | Describe method of data extraction from reports (e.g., piloted forms, independently, in duplicate) and any processes for obtaining and confirming data from investigators. | 7 |
| Data items | 11 | List and define all variables for which data were sought (e.g., PICOS, funding sources) and any assumptions and simplifications made. | 7 |
| **Geometry of the network** | **S1** | Describe methods used to explore the geometry of the treatment network under study and potential biases related to it. This should include how the evidence base has been graphically summarized for presentation, and what characteristics were compiled and used to describe the evidence base to readers. | ***8*** |
| Risk of bias within individual studies | 12 | Describe methods used for assessing risk of bias of individual studies (including specification of whether this was done at the study or outcome level), and how this information is to be used in any data synthesis. | 7 |
| Summary measures | 13 | State the principal summary measures (e.g., risk ratio, difference in means). *Also describe the use of additional summary measures assessed, such as treatment rankings and surface under the cumulative ranking curve (SUCRA) values, as well as modified approaches used to present summary findings from meta-analyses.* | 8 |
| Planned methods of analysis | 14 | Describe the methods of handling data and combining results of studies for each network meta-analysis. This should include, but not be limited to:   - *Handling of multi-arm trials;* - *Selection of variance structure;* - *Selection of prior distributions in Bayesian analyses; and* - *Assessment of model fit.* | 8 |
| **Assessment of Inconsistency** | **S2** | Describe the statistical methods used to evaluate the agreement of direct and indirect evidence in the treatment network(s) studied. Describe efforts taken to address its presence when found. | 10 |
| Risk of bias across studies | 15 | Specify any assessment of risk of bias that may affect the cumulative evidence (e.g., publication bias, selective reporting within studies). | **7** |
| Additional analyses | 16 | Describe methods of additional analyses if done, indicating which were pre-specified. This may include, but not be limited to, the following:   - Sensitivity or subgroup analyses; - Meta-regression analyses; - *Alternative formulations of the treatment network; and* - *Use of alternative prior distributions for Bayesian analyses (if applicable).* | ***8*** |
|  |  |  |  |
| **RESULTS†** |  |  |  |
| Study selection | 17 | Give numbers of studies screened, assessed for eligibility, and included in the review, with reasons for exclusions at each stage, ideally with a flow diagram. | 9 |
| **Presentation of network structure** | **S3** | Provide a network graph of the included studies to enable visualization of the geometry of the treatment network. | ***Figure 2*** |
| **Summary of network geometry** | **S4** | Provide a brief overview of characteristics of the treatment network. This may include commentary on the abundance of trials and randomized patients for the different interventions and pairwise comparisons in the network, gaps of evidence in the treatment network, and potential biases reflected by the network structure. | ***9*** |
| Study characteristics | 18 | For each study, present characteristics for which data were extracted (e.g., study size, PICOS, follow-up period) and provide the citations. | Table 1 |
| Risk of bias within studies | 19 | Present data on risk of bias of each study and, if available, any outcome level assessment. | 9 Table 2 |
| Results of individual studies | 20 | For all outcomes considered (benefits or harms), present, for each study: 1) simple summary data for each intervention group, and 2) effect estimates and confidence intervals. *Modified approaches may be needed to deal with information from larger networks.* | 8 |
| Synthesis of results | 21 | Present results of each meta-analysis done, including confidence/credible intervals. *In larger networks, authors may focus on comparisons versus a particular comparator (e.g. placebo or standard care), with full findings presented in an appendix. League tables and forest plots may be considered to summarize pairwise comparisons.* If additional summary measures were explored (such as treatment rankings), these should also be presented. | ***10-11*** |
| **Exploration for inconsistency** | **S5** | Describe results from investigations of inconsistency. This may include such information as measures of model fit to compare consistency and inconsistency models, *P* values from statistical tests, or summary of inconsistency estimates from different parts of the treatment network. | ***Appendix 2*** |
| Risk of bias across studies | 22 | Present results of any assessment of risk of bias across studies for the evidence base being studied. | 9 Table 2 |
| Results of additional analyses | 23 | Give results of additional analyses, if done (e.g., sensitivity or subgroup analyses, meta-regression analyses*, alternative network geometries studied, alternative choice of prior distributions for Bayesian analyses,* and so forth). | ***10-11*** |
|  |  |  |  |
| **DISCUSSION** |  |  |  |
| Summary of evidence | 24 | Summarize the main findings, including the strength of evidence for each main outcome; consider their relevance to key groups (e.g., healthcare providers, users, and policy-makers). | 12-14 |
| Limitations | 25 | Discuss limitations at study and outcome level (e.g., risk of bias), and at review level (e.g., incomplete retrieval of identified research, reporting bias). *Comment on the validity of the assumptions, such as transitivity and consistency. Comment on any concerns regarding network geometry (e.g., avoidance of certain comparisons).* | 14 |
| Conclusions | 26 | Provide a general interpretation of the results in the context of other evidence, and implications for future research. | 14 |
|  |  |  |  |
| **FUNDING** |  |  |  |
| Funding | 27 | Describe sources of funding for the systematic review and other support (e.g., supply of data); role of funders for the systematic review. This should also include information regarding whether funding has been received from manufacturers of treatments in the network and/or whether some of the authors are content experts with professional conflicts of interest that could affect use of treatments in the network. | ***2*** |

PICOS = population, intervention, comparators, outcomes, study design.

* Text in italics indicateS wording specific to reporting of network meta-analyses that has been added to guidance from the PRISMA statement.

† Authors may wish to plan for use of appendices to present all relevant information in full detail for items in this section.

Appendix 2

Search strategy

PubMed:

| sequence number | content  retrieval |
| --- | --- |
| #1 | acupuncture[Title/Abstract] |
| #2 | acupoint*[Title/Abstract] |
| #3 | moxibustion[Title/Abstract] |
| #4 | "electric stimulation therap*"[Title/Abstract] |
| #5 | "auricular acupuncture"[Title/Abstract] |
| #6 | "transcutaneous electric nerve stimulation"[Title/Abstract] |
| #7 | "transcutaneous electric acupoint"[Title/Abstract] |
| #8 | electroacupuncture[Title/Abstract] |
| #9 | acupressure[Title/Abstract] |
| #10 | "catgut embedding"[Title/Abstract] |
| #11 | "embedding therap*"[Title/Abstract] |
| #12 | #1 OR #2 OR #3 OR #4 OR #5 OR #6 OR #7 OR #8 OR #9 OR #10 OR #11 |
| #13 | acupuncture[MeSH Terms] |
| #14 | "acupuncture therapy"[MeSH Terms] |
| #15 | "acupuncture points"[MeSH Terms] |
| #16 | moxibustion[MeSH Terms] |
| #17 | electroacupuncture[MeSH Terms] |
| #18 | "acupuncture, ear"[MeSH Terms] |
| #19 | "transcutaneous electric nerve stimulation"[MeSH Terms] |
| #20 | "electric stimulation therapy"[MeSH Terms] |
| #21 | #13 OR #14 OR #15 OR #16 OR #17 OR #18 OR #19 OR #20 |
| #22 | #12 OR #21 |
| #23 | "radiation therap*"[Title/Abstract] |
| #24 | radiotherap*[Title/Abstract] |
| #25 | irradiation[Title/Abstract] |
| #26 | mammosite[Title/Abstract] |
| #27 | radiation treatment[Title/Abstract] |
| #28 | brachytherap*[Title/Abstract] |
| #29 | chemoradiotherap*[Title/Abstract] |
| #30 | "x-ray therap*"[Title/Abstract] |
| #31 | radiochemotherap*[Title/Abstract] |
| #32 | radiodermatitis[Title/Abstract] |
| #33 | "radiation-induced oral mucositis"[Title/Abstract] |
| #34 | "radiation pneumonitis"[Title/Abstract] |
| #35 | "radiocystitis"[Title/Abstract] |
| #36 | "radiation proctitis"[Title/Abstract] |
| #37 | "radiation enteritis"[Title/Abstract] |
| #38 | "radiation esophagitis"[Title/Abstract] |
| #39 | "radiation encephalopathy"[Title/Abstract] |
| #40 | "radiation ulcer"[Title/Abstract] |
| #41 | #23 OR #24 OR #25 OR #26 OR #27 OR #28 OR #29 OR #30 OR #31 OR #32 OR #33 OR #34 OR #35 OR #36 OR #37 OR #38 OR #39 OR #40 |
| #42 | radiotherapy[MeSH Terms] |
| #43 | "radiotherapy, computer-assisted"[MeSH Terms] |
| #44 | "radiotherapy, conformal"[MeSH Terms] |
| #45 | chemoradiotherapy[MeSH Terms] |
| #46 | "cranial irradiation"[MeSH Terms] |
| #47 | "heavy ion radiotherapy"[MeSH Terms] |
| #48 | "radiotherapy dosage"[MeSH Terms] |
| #49 | "dose fractionation, radiation"[MeSH Terms] |
| #50 | "radiotherapy, image-guided"[MeSH Terms] |
| #51 | "radiotherapy, high-energy"[MeSH Terms] |
| #52 | "neutron capture therapy"[MeSH Terms] |
| #53 | radiodermatitis[MeSH Terms] |
| #54 | #42 OR #43 OR #44 OR #45 OR #46 OR #47 OR #48 OR #49 OR #50 OR #51 OR #52 OR #53 |
| #55 | #41 OR #54 |
| #56 | #22 AND #55 |

Cochrane:

| sequence number | content  retrieval |
| --- | --- |
| #1 | (acupuncture):ti,ab,kw |
| #2 | (acupoint*):ti,ab,kw |
| #3 | (moxibustion):ti,ab,kw |
| #4 | (electric stimulation therap*):ti,ab,kw |
| #5 | (auricular acupuncture):ti,ab,kw |
| #6 | (transcutaneous electric nerve stimulation):ti,ab,kw |
| #7 | (transcutaneous electric acupoint):ti,ab,kw |
| #8 | (electroacupuncture):ti,ab,kw |
| #9 | (acupressure):ti,ab,kw |
| #10 | (catgut embedding):ti,ab,kw |
| #11 | (embedding therap*):ti,ab,kw |
| #12 | #1 OR #2 OR #3 OR #4 OR #5 OR #6 OR #7 OR #8 OR #9 OR #10 OR #11 |
| #13 | MeSH descriptor:[acupuncture] explore all trees |
| #14 | MeSH descriptor:[acupuncture therapy] explore all trees |
| #15 | MeSH descriptor:[acupuncture points] explore all trees |
| #16 | MeSH descriptor:[moxibustion] explore all trees |
| #17 | MeSH descriptor:[electroacupuncture] explore all trees |
| #18 | MeSH descriptor:[acupuncture, ear] explore all trees |
| #19 | MeSH descriptor:[transcutaneous electric nerve stimulation] explore all trees |
| #20 | MeSH descriptor:[electric stimulation therapy] explore all trees |
| #21 | #13 OR #14 OR #15 OR #16 OR #17 OR #18 OR #19 OR #20 |
| #22 | #12 OR #21 |
| #23 | (radiation therap*):ti,ab,kw |
| #24 | (radiotherap*):ti,ab,kw |
| #25 | (irradiation):ti,ab,kw |
| #26 | (mammosite):ti,ab,kw |
| #27 | (radiation treatment):ti,ab,kw |
| #28 | (brachytherap*):ti,ab,kw |
| #29 | (chemoradiotherap*):ti,ab,kw |
| #30 | (x-ray therap*):ti,ab,kw |
| #31 | (radiochemotherap*):ti,ab,kw |
| #32 | (radiodermatitis):ti,ab,kw |
| #33 | (radiation-induced oral mucositis):ti,ab,kw |
| #34 | (radiation pneumonitis):ti,ab,kw |
| #35 | (radiocystitis):ti,ab,kw |
| #36 | (radiation proctitis):ti,ab,kw |
| #37 | (radiation enteritis):ti,ab,kw |
| #38 | (radiation esophagitis):ti,ab,kw |
| #39 | (radiation encephalopathy):ti,ab,kw |
| #40 | (radiation ulcer):ti,ab,kw |
| #41 | #23 OR #24 OR #25 OR #26 OR #27 OR #28 OR #29 OR #30 OR #31 OR #32 OR #33 OR #34 OR #35 OR #36 OR #37 OR #38 OR #39 OR #40 |
| #42 | MeSH descriptor:[radiotherapy] explore all trees |
| #43 | MeSH descriptor:[radiotherapy, computer-assisted] explore all trees |
| #44 | MeSH descriptor:[radiotherapy, conformal] explore all trees |
| #45 | MeSH descriptor:[chemoradiotherapy] explore all trees |
| #46 | MeSH descriptor:[cranial irradiation] explore all trees |
| #47 | MeSH descriptor:[heavy ion radiotherapy] explore all trees |
| #48 | MeSH descriptor:[radiotherapy dosage] explore all trees |
| #49 | MeSH descriptor:[dose fractionation, radiation] explore all trees |
| #50 | MeSH descriptor:[radiotherapy, image-guided] explore all trees |
| #51 | MeSH descriptor:[radiotherapy, high-energy] explore all trees |
| #52 | MeSH descriptor:[neutron capture therapy] explore all trees |
| #53 | MeSH descriptor:[radiodermatitis] explore all trees |
| #54 | #42 OR #43 OR #44 OR #45 OR #46 OR #47 OR #48 OR #49 OR #50 OR #51 OR #52 OR #53 |
| #55 | #41 OR #54 |
| #56 | #22 AND #55 |

Embase:

| sequence number | content  retrieval |
| --- | --- |
| #1 | ‘acupuncture’:ti,ab,kw |
| #2 | ‘acupoint*’:ti,ab,kw |
| #3 | ‘moxibustion’:ti,ab,kw |
| #4 | ‘electric stimulation therap*’:ti,ab,kw |
| #5 | ‘auricular acupuncture’:ti,ab,kw |
| #6 | ‘transcutaneous electric nerve stimulation’:ti,ab,kw |
| #7 | ‘transcutaneous electric acupoint’:ti,ab,kw |
| #8 | ‘electroacupuncture’:ti,ab,kw |
| #9 | ‘acupressure’:ti,ab,kw |
| #10 | ‘catgut embedding’:ti,ab,kw |
| #11 | ‘embedding therap*’:ti,ab,kw |
| #12 | #1 OR #2 OR #3 OR #4 OR #5 OR #6 OR #7 OR #8 OR #9 OR #10 OR #11 |
| #13 | ‘acupuncture’/exp |
| #14 | ‘acupuncture therapy’/exp |
| #15 | ‘acupuncture points’/exp |
| #16 | ‘moxibustion’/exp |
| #17 | ‘electroacupuncture’/exp |
| #18 | ‘acupuncture, ear’/exp |
| #19 | ‘transcutaneous electric nerve stimulation’/exp |
| #20 | ‘electric stimulation therapy’/exp |
| #21 | #13 OR #14 OR #15 OR #16 OR #17 OR #18 OR #19 OR #20 |
| #22 | #12 OR #21 |
| #23 | ‘radiation therap*’:ti,ab,kw |
| #24 | ‘radiotherap*’:ti,ab,kw |
| #25 | ‘irradiation’:ti,ab,kw |
| #26 | ‘mammosite’:ti,ab,kw |
| #27 | ‘radiation treatment’:ti,ab,kw |
| #28 | ‘brachytherap*’:ti,ab,kw |
| #29 | ‘chemoradiotherap*’:ti,ab,kw |
| #30 | ‘x-ray therap*’:ti,ab,kw |
| #31 | ‘radiochemotherap*’:ti,ab,kw |
| #32 | ‘radiodermatitis’:ti,ab,kw |
| #33 | ‘radiation-induced oral mucositis’:ti,ab,kw |
| #34 | ‘radiation pneumonitis’:ti,ab,kw |
| #35 | ‘radiocystitis’:ti,ab,kw |
| #36 | ‘radiation proctitis’:ti,ab,kw |
| #37 | ‘radiation enteritis’:ti,ab,kw |
| #38 | ‘radiation esophagitis’:ti,ab,kw |
| #39 | ‘radiation encephalopathy’:ti,ab,kw |
| #40 | ‘radiation ulcer’:ti,ab,kw |
| #41 | #23 OR #24 OR #25 OR #26 OR #27 OR #28 OR #29 OR #30 OR #31 OR #32 OR #33 OR #34 OR #35 OR #36 OR #37 OR #38 OR #39 OR #40 |
| #42 | ‘radiotherapy’/exp |
| #43 | ‘radiotherapy, computer-assisted’/exp |
| #44 | ‘radiotherapy, conformal’/exp |
| #45 | ‘chemoradiotherapy’/exp |
| #46 | ‘cranial irradiation’/exp |
| #47 | ‘heavy ion radiotherapy’/exp |
| #48 | ‘radiotherapy dosage’/exp |
| #49 | ‘dose fractionation, radiation’/exp |
| #50 | ‘radiotherapy, image-guided’/exp |
| #51 | ‘radiotherapy, high-energy’/exp |
| #52 | ‘neutron capture therapy’/exp |
| #53 | ‘radiodermatitis’/exp |
| #54 | #42 OR #43 OR #44 OR #45 OR #46 OR #47 OR #48 OR #49 OR #50 OR #51 OR #52 OR #53 |
| #55 | #41 OR #54 |
| #56 | #22 AND #55 |

Web of Science:

| sequence number | content  retrieval |
| --- | --- |
| #1 | Topic: (acupuncture) |
| #2 | Topic: (acupoint) |
| #3 | Topic: (moxibustion) |
| #4 | Topic: (electric stimulation therap*) |
| #5 | Topic: (auricular acupuncture) |
| #6 | Topic: (transcutaneous electric nerve stimulation) |
| #7 | Topic: (transcutaneous electric acupoint) |
| #8 | Topic: (electroacupuncture) |
| #9 | Topic: (acupressure) |
| #10 | Topic: (catgut embedding) |
| #11 | Topic: (embedding therap*) |
| #12 | Topic: (acupuncture therapy) |
| #13 | Topic: (acupuncture points) |
| #14 | Topic: (acupuncture, ear) |
| #15 | #1 OR #2 OR #3 OR #4 OR #5 OR #6 OR #7 OR #8 OR #9 OR #10 OR #11 OR #12 OR#13 OR #14 |
| #16 | Topic: (radiation therap*) |
| #17 | Topic: (radiotherap*) |
| #18 | Topic: (irradiation) |
| #19 | Topic: (mammosite) |
| #20 | Topic: (radiation treatment) |
| #21 | Topic: (brachytherap*) |
| #22 | Topic: (chemoradiotherap*) |
| #23 | Topic: (x-ray therap*) |
| #24 | Topic: (radiochemotherap*) |
| #25 | Topic: (radiodermatitis) |
| #26 | Topic: (radiation-induced oral mucositis) |
| #27 | Topic: (radiation pneumonitis) |
| #28 | Topic: (radiocystitis) |
| #29 | Topic: (radiation proctitis) |
| #30 | Topic: (radiation enteritis) |
| #31 | Topic: (radiation esophagitis) |
| #32 | Topic: (radiation encephalopathy) |
| #33 | Topic: (radiation ulcer) |
| #34 | Topic: (radiotherapy, computer-assisted) |
| #35 | Topic: (radiotherapy, conformal) |
| #36 | Topic: (cranial irradiation) |
| #37 | Topic: (heavy ion radiotherapy) |
| #38 | Topic: (radiotherapy dosage) |
| #39 | Topic: (dose fractionation, radiation) |
| #40 | Topic: (radiotherapy, image-guided) |
| #41 | Topic: (radiotherapy, high-energy) |
| #42 | Topic: (neutron capture therapy) |
| #43 | #16 OR #17 OR #18 OR #19 OR #20 OR #21 OR #22 OR #23 OR #24 OR #25 OR #26 OR #27 OR #28 OR #29 OR #30 OR #31 OR #32 OR #33 OR #34 OR #35 OR #36 OR #37 OR #38 OR #39 OR #40 OR #41 OR #42 |
| #44 | #15 AND #43 |

Ebsco:

| sequence number | content  retrieval |
| --- | --- |
| #1 | SU acupuncture |
| #2 | SU acupoint |
| #3 | SU moxibustion |
| #4 | SU electric stimulation therap* |
| #5 | SU auricular acupuncture |
| #6 | SU transcutaneous electric nerve stimulation |
| #7 | SU transcutaneous electric acupoint |
| #8 | SU electroacupuncture |
| #9 | SU acupressure |
| #10 | SU catgut embedding |
| #11 | SU embedding therap* |
| #12 | SU acupuncture therapy |
| #13 | SU acupuncture points |
| #14 | SU acupuncture, ear |
| #15 | #1 OR #2 OR #3 OR #4 OR #5 OR #6 OR #7 OR #8 OR #9 OR #10 OR #11 OR #12 OR#13 OR #14 |
| #16 | SU radiation therap* |
| #17 | SU radiotherap* |
| #18 | SU irradiation |
| #19 | SU mammosite |
| #20 | SU radiation treatment |
| #21 | SU brachytherap* |
| #22 | SU chemoradiotherap* |
| #23 | SU x-ray therap* |
| #24 | SU radiochemotherap* |
| #25 | SU radiodermatitis |
| #26 | SU radiation-induced oral mucositis |
| #27 | SU radiation pneumonitis |
| #28 | SU radiocystitis |
| #29 | SU radiation proctitis |
| #30 | SU radiation enteritis |
| #31 | SU radiation esophagitis |
| #32 | SU radiation encephalopathy |
| #33 | SU radiation ulcer |
| #34 | SU radiotherapy, computer-assisted |
| #35 | SU radiotherapy, conformal |
| #36 | SU cranial irradiation |
| #37 | SU heavy ion radiotherapy |
| #38 | SU radiotherapy dosage |
| #39 | SU dose fractionation, radiation |
| #40 | SU radiotherapy, image-guided |
| #41 | SU radiotherapy, high-energy |
| #42 | SU neutron capture therapy |
| #43 | #16 OR #17 OR #18 OR #19 OR #20 OR #21 OR #22 OR #23 OR #24 OR #25 OR #26 OR #27 OR #28 OR #29 OR #30 OR #31 OR #32 OR #33 OR #34 OR #35 OR #36 OR #37 OR #38 OR #39 OR #40 OR #41 OR #42 |
| #44 | #15 AND #43 |

CBM:

| sequence number | content  retrieval |
| --- | --- |
| #1 | “acupuncture”[Common field: Intelligence] |
| #2 | “acupoint*”[Common field: Intelligence] |
| #3 | “moxibustion”[Common field: Intelligence] |
| #4 | “electric stimulation therap*”[Common field: Intelligence] |
| #5 | “auricular acupuncture”[Common field: Intelligence] |
| #6 | “transcutaneous electric nerve stimulation”[Common field: Intelligence] |
| #7 | “transcutaneous electric acupoint”[Common field: Intelligence] |
| #8 | “electroacupuncture”[Common field: Intelligence] |
| #9 | “acupressure”[Common field: Intelligence] |
| #10 | “catgut embedding”[Common field: Intelligence] |
| #11 | “embedding therap*”[Common field: Intelligence] |
| #12 | #1 OR #2 OR #3 OR #4 OR #5 OR #6 OR #7 OR #8 OR #9 OR #10 OR #11 |
| #13 | “acupuncture” [Weighted: extended] |
| #14 | “acupuncture therapy” [Weighted: extended] |
| #15 | “acupuncture points” [Weighted: extended] |
| #16 | “moxibustion” [Weighted: extended] |
| #17 | “electroacupuncture” [Weighted: extended] |
| #18 | “acupuncture, ear” [Weighted: extended] |
| #19 | “transcutaneous electric nerve stimulation” [Weighted: extended] |
| #20 | “electric stimulation therapy” [Weighted: extended] |
| #21 | #13 OR #14 OR #15 OR #16 OR #17 OR #18 OR #19 OR #20 |
| #22 | #12 OR #21 |
| #23 | “radiation therap*”[Common field: Intelligence] |
| #24 | “radiotherap*”[Common field: Intelligence] |
| #25 | “irradiation”[Common field: Intelligence] |
| #26 | “mammosite”[Common field: Intelligence] |
| #27 | “radiation treatment”[Common field: Intelligence] |
| #28 | “brachytherap*”[Common field: Intelligence] |
| #29 | “chemoradiotherap*”[Common field: Intelligence] |
| #30 | “x-ray therap*”[Common field: Intelligence] |
| #31 | “radiochemotherap*”[Common field: Intelligence] |
| #32 | “radiodermatitis”[Common field: Intelligence] |
| #33 | “radiation-induced oral mucositis”[Common field: Intelligence] |
| #34 | “radiation pneumonitis”[Common field: Intelligence] |
| #35 | “radiocystitis”[Common field: Intelligence] |
| #36 | “radiation proctitis”[Common field: Intelligence] |
| #37 | “radiation enteritis”[Common field: Intelligence] |
| #38 | “radiation esophagitis”[Common field: Intelligence] |
| #39 | “radiation encephalopathy”[Common field: Intelligence] |
| #40 | “radiation ulcer”[Common field: Intelligence] |
| #41 | #23 OR #24 OR #25 OR #26 OR #27 OR #28 OR #29 OR #30 OR #31 OR #32 OR #33 OR #34 OR #35 OR #36 OR #37 OR #38 OR #39 OR #40 |
| #42 | “radiotherapy” [Weighted: extended] |
| #43 | “radiotherapy, computer-assisted” [Weighted: extended] |
| #44 | “radiotherapy, conformal” [Weighted: extended] |
| #45 | “chemoradiotherapy” [Weighted: extended] |
| #46 | “cranial irradiation” [Weighted: extended] |
| #47 | “heavy ion radiotherapy” [Weighted: extended] |
| #48 | “radiotherapy dosage” [Weighted: extended] |
| #49 | “dose fractionation, radiation” [Weighted: extended] |
| #50 | “radiotherapy, image-guided” [Weighted: extended] |
| #51 | “radiotherapy, high-energy” [Weighted: extended] |
| #52 | “neutron capture therapy” [Weighted: extended] |
| #53 | “radiodermatitis” [Weighted: extended] |
| #54 | #42 OR #43 OR #44 OR #45 OR #46 OR #47 OR #48 OR #49 OR #50 OR #51 OR #52 OR #53 |
| #55 | #41 OR #54 |
| #56 | #22 AND #55 |

VIP:

| sequence number | content  retrieval |
| --- | --- |
| #1 | M=acupuncture |
| #2 | M=acupoint |
| #3 | M=moxibustion |
| #4 | M=electric stimulation therap* |
| #5 | M=auricular acupuncture |
| #6 | M=transcutaneous electric nerve stimulation |
| #7 | M=transcutaneous electric acupoint |
| #8 | M=electroacupuncture |
| #9 | M=acupressure |
| #10 | M=catgut embedding |
| #11 | M=embedding therap* |
| #12 | M=acupuncture therapy |
| #13 | M=acupuncture points |
| #14 | M=acupuncture, ear |
| #15 | #1 OR #2 OR #3 OR #4 OR #5 OR #6 OR #7 OR #8 OR #9 OR #10 OR #11 OR #12 OR#13 OR #14 |
| #16 | M=radiation therap* |
| #17 | M=radiotherap* |
| #18 | M=irradiation |
| #19 | M=mammosite |
| #20 | M=radiation treatment |
| #21 | M=brachytherap* |
| #22 | M=chemoradiotherap* |
| #23 | M=x-ray therap* |
| #24 | M=radiochemotherap* |
| #25 | M=radiodermatitis |
| #26 | M=radiation-induced oral mucositis |
| #27 | M=radiation pneumonitis |
| #28 | M=radiocystitis |
| #29 | M=radiation proctitis |
| #30 | M=radiation enteritis |
| #31 | M=radiation esophagitis |
| #32 | M=radiation encephalopathy |
| #33 | M=radiation ulcer |
| #34 | M=radiotherapy, computer-assisted |
| #35 | M=radiotherapy, conformal |
| #36 | M=cranial irradiation |
| #37 | M=heavy ion radiotherapy |
| #38 | M=radiotherapy dosage |
| #39 | M=dose fractionation, radiation |
| #40 | M=radiotherapy, image-guided |
| #41 | M=radiotherapy, high-energy |
| #42 | M=neutron capture therapy |
| #43 | #16 OR #17 OR #18 OR #19 OR #20 OR #21 OR #22 OR #23 OR #24 OR #25 OR #26 OR #27 OR #28 OR #29 OR #30 OR #31 OR #32 OR #33 OR #34 OR #35 OR #36 OR #37 OR #38 OR #39 OR #40 OR #41 OR #42 |
| #44 | #15 AND #43 |

Wanfang Database:

| sequence number | content  retrieval |
| --- | --- |
| #1 | Subject:(acupuncture) |
| #2 | Subject:(acupoint) |
| #3 | Subject:(moxibustion) |
| #4 | Subject:(electric stimulation therap*) |
| #5 | Subject:(auricular acupuncture) |
| #6 | Subject:(transcutaneous electric nerve stimulation) |
| #7 | Subject:(transcutaneous electric acupoint) |
| #8 | Subject:(electroacupuncture) |
| #9 | Subject:(acupressure) |
| #10 | Subject:(catgut embedding) |
| #11 | Subject:(embedding therap*) |
| #12 | Subject:(acupuncture therapy) |
| #13 | Subject:(acupuncture points) |
| #14 | Subject:(acupuncture, ear) |
| #15 | #1 OR #2 OR #3 OR #4 OR #5 OR #6 OR #7 OR #8 OR #9 OR #10 OR #11 OR #12 OR#13 OR #14 |
| #16 | Subject:(radiation therap*) |
| #17 | Subject:(radiotherap*) |
| #18 | Subject:(irradiation) |
| #19 | Subject:(mammosite) |
| #20 | Subject:(radiation treatment) |
| #21 | Subject:(brachytherap*) |
| #22 | Subject:(chemoradiotherap*) |
| #23 | Subject:(x-ray therap*) |
| #24 | Subject:(radiochemotherap*) |
| #25 | Subject:(radiodermatitis) |
| #26 | Subject:(radiation-induced oral mucositis) |
| #27 | Subject:(radiation pneumonitis) |
| #28 | Subject:(radiocystitis) |
| #29 | Subject:(radiation proctitis) |
| #30 | Subject:(radiation enteritis) |
| #31 | Subject:(radiation esophagitis) |
| #32 | Subject:(radiation encephalopathy) |
| #33 | Subject:(radiation ulcer) |
| #34 | Subject:(radiotherapy, computer-assisted) |
| #35 | Subject:(radiotherapy, conformal) |
| #36 | Subject:(cranial irradiation) |
| #37 | Subject:(heavy ion radiotherapy) |
| #38 | Subject:(radiotherapy dosage) |
| #39 | Subject:(dose fractionation, radiation) |
| #40 | Subject:(radiotherapy, image-guided) |
| #41 | Subject:(radiotherapy, high-energy) |
| #42 | Subject:(neutron capture therapy) |
| #43 | #16 OR #17 OR #18 OR #19 OR #20 OR #21 OR #22 OR #23 OR #24 OR #25 OR #26 OR #27 OR #28 OR #29 OR #30 OR #31 OR #32 OR #33 OR #34 OR #35 OR #36 OR #37 OR #38 OR #39 OR #40 OR #41 OR #42 |
| #44 | #15 AND #43 |

CNKI:

| sequence number | content  retrieval |
| --- | --- |
| #1 | ti,kw,ab=acupuncture |
| #2 | ti,kw,ab=acupoint |
| #3 | ti,kw,ab=moxibustion |
| #4 | ti,kw,ab=electric stimulation therap* |
| #5 | ti,kw,ab=auricular acupuncture |
| #6 | ti,kw,ab=transcutaneous electric nerve stimulation |
| #7 | ti,kw,ab=transcutaneous electric acupoint |
| #8 | ti,kw,ab=electroacupuncture |
| #9 | ti,kw,ab=acupressure |
| #10 | ti,kw,ab=catgut embedding |
| #11 | ti,kw,ab=embedding therap* |
| #12 | ti,kw,ab=acupuncture therapy |
| #13 | ti,kw,ab=acupuncture points |
| #14 | ti,kw,ab=acupuncture, ear |
| #15 | #1 OR #2 OR #3 OR #4 OR #5 OR #6 OR #7 OR #8 OR #9 OR #10 OR #11 OR #12 OR#13 OR #14 |
| #16 | ti,kw,ab=radiation therap* |
| #17 | ti,kw,ab=radiotherap* |
| #18 | ti,kw,ab=irradiation |
| #19 | ti,kw,ab=mammosite |
| #20 | ti,kw,ab=radiation treatment |
| #21 | ti,kw,ab=brachytherap* |
| #22 | ti,kw,ab=chemoradiotherap* |
| #23 | ti,kw,ab=x-ray therap* |
| #24 | ti,kw,ab=radiochemotherap* |
| #25 | ti,kw,ab=radiodermatitis |
| #26 | ti,kw,ab=radiation-induced oral mucositis |
| #27 | ti,kw,ab=radiation pneumonitis |
| #28 | ti,kw,ab=radiocystitis |
| #29 | ti,kw,ab=radiation proctitis |
| #30 | ti,kw,ab=radiation enteritis |
| #31 | ti,kw,ab=radiation esophagitis |
| #32 | ti,kw,ab=radiation encephalopathy |
| #33 | ti,kw,ab=radiation ulcer |
| #34 | ti,kw,ab=radiotherapy, computer-assisted |
| #35 | ti,kw,ab=radiotherapy, conformal |
| #36 | ti,kw,ab=cranial irradiation |
| #37 | ti,kw,ab=heavy ion radiotherapy |
| #38 | ti,kw,ab=radiotherapy dosage |
| #39 | ti,kw,ab=dose fractionation, radiation |
| #40 | ti,kw,ab=radiotherapy, image-guided |
| #41 | ti,kw,ab=radiotherapy, high-energy |
| #42 | ti,kw,ab=neutron capture therapy |
| #43 | #16 OR #17 OR #18 OR #19 OR #20 OR #21 OR #22 OR #23 OR #24 OR #25 OR #26 OR #27 OR #28 OR #29 OR #30 OR #31 OR #32 OR #33 OR #34 OR #35 OR #36 OR #37 OR #38 OR #39 OR #40 OR #41 OR #42 |
| #44 | #15 AND #43 |

Appendix 3

Reference of included studies.

1. Lei M, Chen H, Hao B. Clinical Effect and Safety Study of Guchang Zhixie Prescription Combined with Shu-Mu Acupoint Catgut in the Treatment of Radiation Proctitis. Sichuan Journal of Traditional Chinese Medicine. 2021;39(2).

2. Pan S, Zhou Y. Clinical Observation of Mild Moxibustion Combined with Western Medication for Radiation Enteritis. Shanghai Journal of Acupuncture and Moxibustion. 2021;40(6). doi:10.13460/j.issn.1005-0957.2021.06.0676.

3. Wang Q. The Efficacy of Warm Moxibustion Package for Point Hot Compress in the Treatment of Radiation Proctitis of Cervix Cancer Patients. Chinese Journal of Coloproctology. 2021;41(2). doi:10.3969/j.issn.1000-1174.2021.02.018.

4. Xie Y. Effect of Acupoint Injection Combined with Retention Enema of Yuchang Decoction in the Treatment of Acute Radiation Proctitis. Journal of Colorectal and Anal Surgery. 2021;27(S2). doi:10.19668/j.cnki.issn1674-0491.2021.S2.0001.

5. Fang W. Clinical Observation on the Effect of Moxibustion Combined with Retention Enema in the Treatment of Intestinal Response after Radiotherapy in Patients with Pelvic Tumor. Tibetan Medicine. 2020;41(1). doi:CNKI:SUN:XZYY.0.2020-01-077.

6. Dong D. Clinical Observation on the Effect of Acupuncture Combined with Medication in the Treatment of Damp-heat Radiation Proctitis. Zhejiang Clin Med. 2020;22(5).

7. Li M, Wang L, Wang J, Sun P, Fei D, Wang L, Zhou T. Clinical Observation on the Effect of Traditional Chinese Medicine Characteristic Nursing Intervention on Hemorrhagic Radiation Enteritis. World Latest Medicine Information. 2019;19(28). doi:10.19613/j.cnki.1671-3141.2019.28.172.

8. Yang Y. Clinical Effect of the Back-Shu and Front-Mu Acupoints Embedding Treatment for Chronic Radioactive Enteritis. Chinese And Foreign Medical Research. 2019;17(30). doi:10.14033/j.cnki.cfmr.2019.30.023.

9. Zhang Q, Ding H, Han T, Li Z. Clinical Treatment of Radiation-induced Enteritis in Patients with Pelvic Tumor. Chineses Journal of Urban And Rural Enterprise Hygine. 2019;(9). doi:10.16286/j.1003-5052.2019.09.067.

10. Zhong F, Yan H, Bi L, Bai C. Effect Observation of Acupoint Injection Combined with Chinese Medicine Retention Enema on Acute Radiation Proctitis. Liaoning Journal of Traditional Chinese Medicine. 2019;46(7). doi:10.13192/j.issn.1000-1719.2019.07.048.

11. Luo T, Meng Z. The Clinical Research into Chronic Radiation Enteritis Treated with “Shu-Mu-Point Combination” Catgut Embedding. Henan Traditional Chinese Medicine. 2018;38(11). doi:10.16367/j.issn.1003-5028.2018.11.0470.

12. Chen A, Qiu S, Gao L, Xia Y, Liang Z. Effect Observation of Abdominal Moxibustion on Radiation Enteritis in Cervical Cancer. Jouranl of Nurses Training. 2017;32(21). doi:10.16821/j.cnki.hsjx.2017.21.015.

13. Chen T. Effect of Acupuncture Combined with Drug Retention Enema in the Treatment of Long-term Radiation Enteritis of Cervical Cancer. Chinese Practical Medicine. 2016;11(1). doi:10.14163/j.cnki.11-5547/r.2016.01.143.

14. Wu X, Wu H, Yao J, Li J, Zhang X, Zhou J. Effect of acupoint hot compress with warming moxibustion bag on radiation proctitis caused by radiotherapy in cervical cancer. Chinese Journal of Practical Nursing. 2016;32(34). doi:10.3760/cma.j.issn.1672-7088.2016.34.011.

15. Long Z, Wang B. Clinical observation on 31 cases of radiation proctitis treated by TCM. Chinese Journal of Integrated Traditional and Western Medicine. 2015;23(3). doi:10.3969/j.issn.1671-038X.2015.03.15.

16. Qiu S, Kong Y, Liang Z, Li Q, Gui L, Yi M, Xia Y, Gao L. Effect of moxibustion in treatment of acute radiation proctitis in patients with cervical cancer. Guangdong Medicial Journal. 2015;36(6). doi:10.13820/j.cnki.gdyx.20150330.012.

17. Yue X. Efficacy of Traditional Chinese Medicine Retention Enema with Acupoint Moxibustion in the Treatment of Radiation Enteritis. China Health Standard Management. 2015;6(28). doi:10.3969/j.issn.1674-9316.2015.28.113.

18. Zhu Y, Li R. Clinical Observation on 23 Cases of Radiation Enteritis by Acupoint Application Combined with Herbal Retention Enema. Yunnan Journal of Traditional Chinese Medicine and Materia Medica. 2015;36(3). doi:10.16254/j.cnki.53-1120/r.2015.03.021.

19. Qiu G, Zhou M, Jiao Q. Clinical efficacy of TCM retention enema combined with moxibustion and massage therapy in the treatment of radiation enteritis. International Medicine and Health Guidance News. 2014;20(2). doi:10.3760/cma.j.issn.1007-1245.2014.02.017.

20. Lin H, Fan Z, Yue S, Zhang L. Observation of Retention Enema of Traditional Chinese Medicine Combined with Moxibustion Treatment on Radiation Enteritis. Hebei Journal of Traditional Chinese Medicine. 2013;35(6). doi:10.3969/j.issn.1002-2619.2013.06.007.

21. Ji R, Chen Z. Effect of Acupuncture Combined with Drug Retention Enema in the Treatment of Long-term Radiation Enteritis of Cervical Cancer. Medical Journal of Communications. 2008;22(6). doi:10.3969/j.issn.1006-2440.2008.06.066.

22. Li Y, Ma C. Therapeutic Effect of Acupuncture Combined with Imodium on 30 Cases of Radiation Enteritis. Hebei Journal of Traditional Chinese Medicine. 2007;(2). doi:10.3969/j.issn.1002-2619.2007.02.040.

23. Yang J, Chen G, Yu M, Fu L, Deng X, Fan F, Zhang Y, Zhao R, Liu J. Clinical Research on Acupuncture Treatment of Radiation Proctitis and Radiation Cystitis. Chinese Acupuncture and Moxibustion. 1994;14(4). doi:10.3321/j.issn:0255-2930.1994.04.010.

24. Xie L, Diao B, Diao C, Han L, Liu D, Liu H. Effects of Fuzheng Jiedu Kang'ai Decoction Combined with Moxibustion in Treatment of NSCLC after Radiotherapy. Liaoning Journal of Traditional Chinese Medicine. 2016;43(4). doi:10.13192/j.issn.1000-1719.2016.04.033.

25. Zheng K, Deng T, Liu Z, Dong B. Clinical Observation on Advanced Esophageal Carcinoma Treated by Combined Use of Acupuncture plus Drug and Radiotherapy. Journal of Liaoning University of Traditional Chinese Medicine. 2014;16(10). doi:10.13194/j.issn.1673-842x.2014.10.003.

26. Ge Y. Influence of Acupoint Application on Leukocyte in Patients with Radiotherapy. Journal of Clinical Medicine in Practice. 2012;16(20). doi:CNKI:SUN:XYZL.0.2012-20-008.

27. Zhu D, Wu Y, Shen H, Xu Z, Zhang T. Acupoint Application in the Prevention of Myelosuppression Induced by Radiotherapy. Chinese Journal of Radiological Health. 2009;18(4). doi:CNKI:SUN:REDI.0.2009-04-068.

28. Zhang L, Zhou L. Observation and Nursing Care of Point Massage for the Prophylaxis of Leucocytopenia from Radiotherapy. Chinese Journal of Medicine and Nursing. 2007;4(9).

29. Sun Y. Exploring the Effects of Moxibustion on Yongquan Acupoint in Prevention and Treatment of Acute Oral Mucosal Reactions after Radiotherapy of Head and Neck Neoplasms. World Latest Medicine Information. 2019;19(69). doi:10.19613/j.cnki.1671-3141.2019.69.001.

30. Liang K. Acupuncture combined Yiqiyangyin Decoction Treatment Adverse Radiotherapy Randomized Parallel Group Study. Journal of Practical Traditional Chinese Internal Medicine. 2015;29(8). doi:10.13729/j.issn.1671-7813.2015.08.64.

31. Wang F, Wang Y. Clinical Effects of Acupuncture and Moxibustion Therapy plus Chinese Herbal Preparation in Nasopharyngeal Carcinoma Patients Undergone Radiotherapy. Modern Chinese Doctor. 2012;50(28). doi:CNKI:SUN:ZDYS.0.2012-28-039.

32. Zhong M. Clinical Observation on the Prevention of Acute Radiation Injury in Patients with Nasopharyngeal Carcinoma. Journal of Emergency in Traditional Chinese Medicine. 2012;21(10). doi:10.3969/j.issn.1004-745X.2012.10.054.

33. Liu G. Herbal Acupoint Application in the Treatment of Oral Mucosa Injury During Radiotherapy for Nasopharyngeal Carcinoma. Journal of North China Coal Medical College. 2002;4(4). doi:10.3969/j.issn.1008-6633.2002.04.075.

34. Li S, Lin G, Wang B, Zhou T. Clinical Observation of Herbal Acupoint Application in Preventing Oral Mucosal Reaction During Radiotherapy for Nasopharyngeal Carcinoma. Chinese Journal of Nursing. 1999;34(10). doi:10.3321/j.issn:0254-1769.1999.10.020.

35. Huang Y. Treatment of 50 cases on Buxin Decoction Combined with KI10 Acupoint Massage for xerostomia after radiotherapy. Zhejiang Journal of Traditional Chinese Medicine. 2020;55(6). doi:10.3969/j.issn.0411-8421.2020.06.027.

36. Dalbem PÉ, Costa BBM, Gonzales ZV, Beatris MV, Edler MF. Effects of transcutaneous electrical nerve stimulation on the salivary flow of patients with hyposalivation induced by radiotherapy in the head and neck region-A randomised clinical trial. Journal of oral rehabilitation. 2019;46(12). doi:10.1111/joor.12851.

37. Wong RK, Deshmukh S, Wyatt G, Sagar S, Singh AK, Sultanem K, Nguyen-Tân PF, Yom SS, Cardinale J, Yao M, Hodson I, Matthiesen CL, Suh J, Thakrar H, Pugh SL, Berk L. Acupuncture-Like Transcutaneous Electrical Nerve Stimulation Versus Pilocarpine in Treating Radiation-Induced Xerostomia: Results of RTOG 0537 Phase 3 Study. International journal of radiation oncology, biology, physics. 2015;92(2). doi:10.1016/j.ijrobp.2015.01.050.

38. Meng Z, Garcia MK, Hu C, Chiang J, Chambers M, Rosenthal DI, Peng H, Zhang Y, Zhao Q, Zhao G, Liu L, Spelman A, Palmer JL, Wei Q, Cohen L. Randomized controlled trial of acupuncture for prevention of radiation-induced xerostomia among patients with nasopharyngeal carcinoma. Cancer. 2012;118(13). doi:10.1002/cncr.26550.

39. Meng Z, Kay GM, Hu C, Chiang J, Chambers M, Rosenthal DI, Peng H, Wu C, Zhao Q, Zhao G, Liu L, Spelman A, Lynn PJ, Wei Q, Cohen L. Sham-controlled, randomised, feasibility trial of acupuncture for prevention of radiation-induced xerostomia among patients with nasopharyngeal carcinoma. European journal of cancer (Oxford, England : 1990). 2012;48(11). doi:10.1016/j.ejca.2011.12.030.

40. Braga FP, Lemos JCA, Alves FA, Migliari DA. Acupuncture for the prevention of radiation-induced xerostomia in patients with head and neck cancer. Brazilian oral research. 2011;25(2). doi:10.1590/s1806-83242011000200014.

41. Blom M, Dawidson I, Fernberg JO, Johnson G, Angmar-Månsson B. Acupuncture treatment of patients with radiation-induced xerostomia. Eur J Cancer B Oral Oncol. 1996;32B(3). doi:10.1016/0964-1955(95)00085-2.
